# Multimodal Fusion of Echocardiogram Images and Electronic Medical Records for Heart Disease Screening: Retrospective Algorithm Development and Validation Study

**DOI:** 10.2196/78949

**Published:** 2026-05-14

**Authors:** Bokai Yang, Yirong Qin, Ye Li, Ruitao Xie, Limai Jiang, Juan He, Jikui Liu, Yunpeng Cai

**Affiliations:** 1 Institute of Intelligence Science and Engineering, Shenzhen Polytechnic University, No. 4089 Shahe West Road, Shenzhen, China; 2 Shenzhen Institute of Advanced Technology, Chinese Academy of Sciences, 1068 Xueyuan Avenue, Shenzhen University Town, Shenzhen, 518000, China; 3 Suzhou City University, Suzhou, China; 4 University of Chinese Academy of Sciences, Beijing, China; 5 Department of Computer and Information Science, Faculty of Science and Technology, University of Macau, Macau, China

**Keywords:** electronic medical record, echocardiogram images, convolutional neural network, multimodal, heart disease classification

## Abstract

**Background:**

Echocardiography is a fundamental imaging modality for the diagnosis of heart disease (HD), but its interpretation remains operator-dependent and lacks standardized, data-driven decision support. Although artificial intelligence has improved image-based diagnosis, the added value and interpretability of integrating routinely collected electronic medical records (EMRs) with echocardiogram (ECHO) for large-scale screening remain underexplored.

**Objective:**

This study aims to develop an explainable artificial intelligence framework that integrates multimodal data, including ECHO images and EMRs, with derived temporal and clinical features, to enhance the screening and interpretability of heart disease diagnosis.

**Methods:**

In this retrospective single-center study, we analyzed 26,936 patients with linked ECHO and EMR records (1470 with clinically confirmed HD and 25,466 without). We constructed cross-sectional EMR features (demographics, comorbidities, and registration history) and extracted view-specific image features with a simplified Inception-v3 backbone. Five modality-specific feature vectors (4 imaging views + EMR) were concatenated and input to an extreme gradient boosting classifier within a patient-level stratified 5-fold cross-validation framework. To mitigate class imbalance during training, non-HD cases in the training folds were randomly downsampled to a 1:3 HD:non-HD ratio; held-out test folds preserved the original prevalence. Post hoc explainability was provided via Grad-CAM heatmaps for images and Shapley additive explanations analysis for EMR features.

**Results:**

For the primary binary task (presence vs absence of HD), the multimodal model achieved an AUC of 0.8147 (SD 0.009) on held-out test folds, compared with 0.7785 (SD 0.014) for the image-only baseline, and 0.7343 (SD 0.009) for the EMR-only baseline. At the clinically selected decision threshold, sensitivity, specificity, positive predictive value, and negative predictive value were 84.6% (SD 1.6%), 72.4% (SD 2%), 15% (SD 2.3%), 98.8% (SD 0.3%) for the multimodal model, versus 82.3% (SD 1.9%), 73.5% (SD 2%), 15.2% (SD 2.1%), 98.6% (SD 0.3%) (image-only), and 77.5% (SD 2.1%), 67% (SD 2.3%), 11.9% (SD 1.7%), 98.1% (SD 0.4%) (EMR-only). Grad-CAM visualizations qualitatively highlighted anatomically and physiologically meaningful regions (eg, valvular structures and abnormal flow jets), while Shapley additive explanations analysis identified age, registration years, sex, and hypertension among the top EMR contributors—findings that align with established cardiovascular risk factors.

**Conclusions:**

Integrating echocardiographic images and EMR data in an explainable multimodal framework yields improved and clinically plausible screening performance for heart disease. Future work should focus on external multicenter validation, quantitative assessment of visual explanations against expert annotations, and prospective studies assessing clinical use and workflow integration.

## Introduction

Heart disease (HD) continues to be one of the leading causes of morbidity and mortality worldwide, placing a substantial burden on both public health systems and individual quality of life [1]. Early identification and risk stratification remain essential for improving clinical outcomes [2]. Transthoracic echocardiography (ECHO) is regarded as the primary imaging modality for noninvasive cardiac evaluation because of its accessibility, safety, and cost-effectiveness. Despite its widespread use, ECHO interpretation is inherently operator-dependent, and diagnostic accuracy varies with clinical expertise, institutional workflow, and patient-specific factors [3]. These challenges underscore the need for standardized, data-driven decision-support tools that can enhance the consistency and accuracy of ECHO-based diagnosis.

Traditional cardiovascular risk prediction models and classical machine learning (ML) approaches have been applied to assist HD assessment [4,5]. However, such models typically rely on handcrafted features, limited physiological assumptions, or population-specific parameters, which restrict their generalizability in diverse clinical environments [6-10]. Moreover, these models often treat imaging and clinical variables independently, overlooking the potential complementary value of integrating the 2 modalities [11].

Recent advancements in deep learning have greatly expanded the capabilities of automated ECHO analysis, enabling applications such as view classification, cardiac chamber quantification, functional assessment, and disease detection [12-20]. While these imaging-based artificial intelligence (AI) models have demonstrated promising results, most rely solely on pixel-level information and do not incorporate routinely collected electronic medical records (EMRs), which contain valuable demographic, temporal, and comorbidity-related data pertinent to cardiac risk prediction [21,22]. Several multimodal approaches have been explored, but existing studies are often limited by small sample sizes, dataset heterogeneity, incomplete fusion strategies, or insufficient evaluation protocols [23-39]. Furthermore, explainability remains a major barrier to clinical implementation: many prior models provide limited insight into how predictions are derived, reducing their transparency and acceptance among clinicians.

Despite growing interest in multimodal learning, there remains a notable gap: no prior large-scale study has developed and rigorously evaluated an explainable framework that fuses 4-view transthoracic ECHO with structured EMR features for comprehensive HD screening. Existing works typically lack patient-level validation across large real-world cohorts, rely on single-modality inputs, or provide inadequate interpretability. These limitations hinder their practical deployment in clinical decision support.

To address these gaps, this study makes three key contributions:

1. We propose a novel explainable multimodal AI framework that integrates ECHO images from 4 standard views with structured EMR-derived features, leveraging complementary information from both modalities for enhanced HD screening.
2. We perform large-scale, patient-level stratified 5-fold cross-validation on a real-world clinical cohort of 26,936 individuals (1470 with HD and 25,466 without), ensuring robust performance assessment under realistic class imbalance and eliminating patient-level data leakage.
3. We incorporate modality-specific interpretability, combining Grad-CAM for visualizing image-based attention patterns with Shapley additive explanations (SHAP) analysis for quantifying EMR feature contributions. This dual-level explainability provides clinically meaningful insights and increases transparency, supporting future integration into routine diagnostic workflows.

Overall, this study advances the development of clinically informed multimodal diagnostic models by unifying echocardiographic and EMR data in an interpretable framework. The results demonstrate the potential of explainable AI to enhance screening accuracy, reduce operator dependency, and strengthen clinician trust in machine-assisted cardiac assessment.

## Methods

### Material

The EMRs data includes a total of 27,398 individuals, out of which 26,936 individuals have corresponding ECHO imaging data, and 462 individuals do not have corresponding ECHO imaging data. All EMR data were recorded prior to the last ECHO examination. The index date was defined as each patient’s last echocardiographic examination. EMR variables were extracted from the entire history prior to (and including) the index date. Binary clinical history and symptom variables (eg, hypertension, diabetes mellitus [DM], atrial fibrillation [AF], and chest tightness or pain [Cpain]) were coded as 1 if ever recorded before the index date and 0 otherwise (including absence of documentation). Among the 26,936 individuals with corresponding ECHO imaging, 1470 individuals have HD, and 25,466 individuals do not have HD. HD labels were identified from the EMRs using either free-text discharge diagnoses or the corresponding ICD-10 codes. HD in this context refers to both structural and functional abnormalities of the heart, encompassing conditions such as congenital heart defects, rheumatic and pulmonary HDs, nonischemic cardiomyopathy, and valvular disorders involving the mitral, tricuspid, aortic, and pulmonary valves. It also includes ventricular hypertrophy, heart failure, hypertensive HD, as well as septal anomalies like ventricular and atrial septal defects. HD refers to structural and functional disorders of the heart, broadly categorized into 3 types. The first category pertains to valvular and hemodynamic abnormalities, such as congenital heart defects, rheumatic heart conditions, and diseases affecting the heart valves. The second category includes disorders related to ventricular structure and function, including pulmonary HD, nonischemic cardiomyopathy, and hypertensive cardiac conditions. The third type is heart failure.

The field list of the EMRs is shown in Table 1, and the distribution and numbers of patients with HD subtypes in the study cohort are shown in Table 2. For Table 2, HD subtypes were counted in a non–mutually exclusive manner because multiple subtypes can be recorded for the same patient within a category. For the multiclass (3-class and 4-class) tasks, we used the top-level category as the ground-truth label, and these categories were constructed to be mutually exclusive at the patient level (ie, no patient had diagnoses spanning more than one category).

**Table 1.** Explanation of the EMRs^a^ data fields used in this study. This table summarizes the EMR variables extracted from a retrospective single-center cohort of individuals undergoing routine transthoracic echocardiographic examinations for heart disease screening at a medical institution in China.

List of fields	Explanation	Notes
Pid	Resident personal identification	Consistent with the Pid in cardiac ECHO^b^ and can be correlated
HTN	Hypertension	0 No 1 Yes
DM	Diabetes	0 No 1 Yes
Hlipid	Hyperlipidemia	0 No 1 Yes
Huric	Hyperuricemia	0 No 1 Yes
AR	Arrhythmias	Tachycardia, bradycardia, asystole, conduction block, and flutter
AF	Atrial fibrillation	0 No 1 Yes
PB	Premature beat	Atrial premature beats, ventricular premature beats, and sinus premature beats
Cpain	Chest tightness or pain	0 No 1 Yes
Hache	Headache or dizziness	0 No 1 Yes
Sex_code	Gender code	0 male and 1 female
Date_of_birth	Date of birth	—
Reg_date	Image examination registration date	Reg_date

a EMRs: electronic medical records.

b ECHO: echocardiogram.

**Table 2.** Distribution and numbers of patients with heart disease subtypes in the study cohort.

Disease category	Subtype	Number of patients, n
Abnormalities of heart valves and blood flow	Congenital HD^a^	451
	Rheumatic HD	193
	Valvular HD	340
Ventricular abnormalities	Pulmonary HD	66
	Cardiomyopathy	283
	Hypertensive HD	43
Heart failure	Heart failure	181

a HD: heart disease.

**Figure 1.**
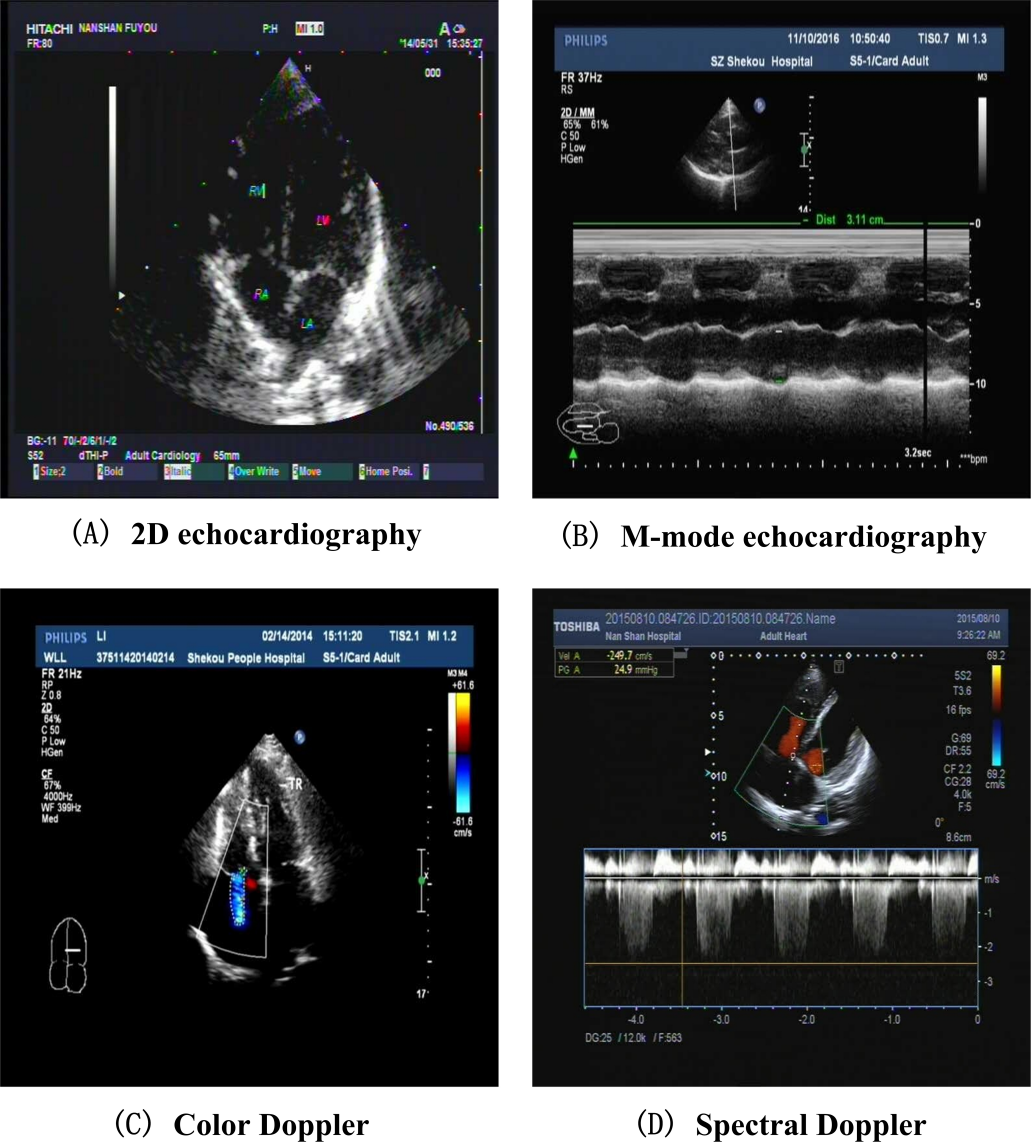
The four main categories of echocardiogram images in this study. Examples are shown from a retrospective single-center cohort of individuals undergoing routine cardiac examinations at a medical institution in China. The four panels illustrate the main echocardiographic modalities analyzed in this work: (A) 2D echocardiography, (B) M-mode echocardiography, (C) color Doppler imaging, and (D) spectral Doppler imaging, which together provide structural and blood-flow information for automated heart disease screening.

The ECHO imaging data comprises 27,398 individuals, with a total of 32,111 ECHO examinations and 530,569 images. Among the 27,398 individuals, 26,936 individuals have corresponding EMR data, and 462 individuals do not have corresponding EMR data. The field list for the ECHO imaging data is detailed in the following table. The ECHO imaging data can be divided into 4 categories, as shown in Figure 1, namely 2D echocardiography, M-mode echocardiography, color Doppler, and spectral Doppler.

### Ethical Considerations

Data used in this study were obtained from Huazhong University of Science and Technology Union Hospital (Nanshan Hospital), China. Medical imaging data and EMRs were extracted from the hospital Picture Archiving and Communication System and Hospital Information System, respectively, and were linked across systems for patients with the same Pid. This study was conducted as a retrospective analysis of routinely collected clinical data. Given that all medical records and echocardiogram images were acquired during standard clinical procedures and fully anonymized prior to analysis, a waiver of written informed consent was granted in accordance with Clause 32 of the World Medical Association’s Declaration of Helsinki. The study protocol received approval from the Institutional Review Board of the Shenzhen Institute of Advanced Technology, Chinese Academy of Sciences (Approval No. SIAT-IRB-151115-H0084).

All direct personal identifiers (eg, name, medical record number, contact information, and exact dates of birth) were removed or replaced with study-specific codes before data extraction, and the de-identified dataset was stored on secure servers with access restricted to authorized investigators only. As this work involved secondary analysis of existing de-identified records and required no additional visits or interventions, participants did not receive any financial or other compensation. All echocardiographic images and example screenshots presented in the manuscript and Multimedia Appendix 1 were carefully inspected to ensure that no individual patient can be identified; any potentially identifying overlays present in the original images were masked or removed prior to inclusion.

### Algorithm

The aim of this study is to classify HD through the multimodal fusion of EMRs and medical imaging data. Specifically, we preprocess the EMRs and medical imaging data and then feed the processed data into a multimodal model for training. It’s worth noting that our multimodal framework is implemented as a staged pipeline, where the image encoder and the downstream fusion, classifier components are trained sequentially rather than jointly. For the imaging input, we use a simplified version of Inception_V3 with specific layers removed. Specifically, our model follows the canonical Inception-V3 network in the stem, Inception-A, BC, and reduction blocks, but removes the last 2 Inception Module C blocks in the final grid. Apart from this truncation and the adaptation of the final fully connected layer to match the number of target classes, all convolutional kernel sizes, strides, and filter numbers are kept identical to the original Inception-V3 implementation. Our model architecture incorporates 3 standard Inception modules operating at a 35×35 resolution, each with 288 filters. A grid reduction operation subsequently compresses this to a 17×17 resolution with 768 filters. The output dimension of each module serves as the input to the following one. To preserve spatial dimensions, zero-padding is applied to convolutional layers that maintain grid size, including those within non-downsampling Inception modules; other layers, particularly those performing downsampling, omit padding. The simplified Inception-V3 network was trained using the Adam optimizer with an initial learning rate of 0.001 and binary cross-entropy loss, using a mini-batch size of 32. Input echocardiogram frames were resized to 299×299×3 to match the network input. Training was run for up to 1024 epochs with early stopping based on the validation loss (patience=10 epochs), and the model achieving the lowest validation loss on the validation set was selected for evaluation. Unless otherwise specified, all other optimization hyperparameters followed the default settings of the TensorFlow Keras implementation of Inception-V3 and Adam.

For each image type, we use a pretrained Inception-v3 network as the initialization point. Fine-tuning this model, rather than training from scratch, significantly improves efficiency and convergence. The Inception-v3 model, originally trained on the ImageNet dataset—which classifies over a million natural images into 1000 categories—is adopted here via transfer learning. A streamlined version of Inception-v3 is used for feature extraction, where selected inception blocks are removed to simplify the network. This simplification was motivated by the moderate size of our dataset and preliminary experiments, in which the full-depth Inception-V3 showed signs of overfitting without improving validation performance, whereas the truncated variant achieved comparable discrimination with fewer parameters and reduced training time. For each of the 4 echocardiographic views (2D, M-mode, color Doppler, and spectral Doppler), the simplified Inception-v3 backbone processes the input images and outputs a fixed-length feature vector, denoted as $f^{\left(2D\right)},f^{\left(M\right)},f^{\left(CD\right)}$ and $f^{\left(SD\right)}$, respectively. The EMR modality is represented by a 12-dimensional vector $f^{\left(EMR\right)}$ containing the normalized clinical variables described in Table 1 (for example, hypertension, diabetes, hyperlipidemia, age, and registration years). Feature fusion is implemented as intermediate (feature-level) fusion by concatenating these five modality-specific feature vectors into a single fused representation:


$$z=\left[f^{\left(2D\right)};f^{\left(M\right)};f^{\left(CD\right)};f^{\left(SD\right)};f^{\left(EMR\right)}\right]$$

The fused feature vector z is used as the input to an extreme gradient boosting (XGBoost) classifier. Specifically, we first train and fine-tune the simplified Inception-v3 backbones to obtain fixed-length image embeddings. These embeddings are then concatenated with EMR features to train the XGBoost classifier; during XGBoost training, the image backbone is kept fixed (ie, no joint backpropagation across modules). The main hyperparameters were set as follows: learning rate (eta)=0.01, maximum tree depth=4, evaluation metric = “AUC,” subsample ratio=0.75, column sampling rate for each tree (colsample_bytree)=0.75, number of threads=8, random seed=42, and silent mode enabled. All other hyperparameters used the default values of the XGBoost library. For binary classification tasks, we use XGBoost with a logistic (“binary: logistic”) objective to obtain the probability of HD. For multiclass tasks with C classes, we use the “multi:softprob” objective to produce a C-dimensional probability vector, and the predicted class is obtained $arg\;max_{cPc}$. All reported receiver operating characteristic curves and area under the receiver operating characteristic curve (AUC) values are computed from these XGBoost output probabilities.

### Experimental Setup

#### Experimental Environment

All experiments were conducted using TensorFlow (version 2.3.1), a widely adopted deep learning framework, on a system running the Windows Server 2012 operating system. The computational environment consisted of a high-performance server featuring 8-core Intel Xeon E5-2690 V4 CPUs and 64 GB of RAM. Additionally, the server was equipped with an NVIDIA Tesla P100 GPU with 16 GB of dedicated memory to accelerate model training. Python 3.6 was used as the primary programming language throughout the implementation.

#### Experimental Framework

**Figure 2.**
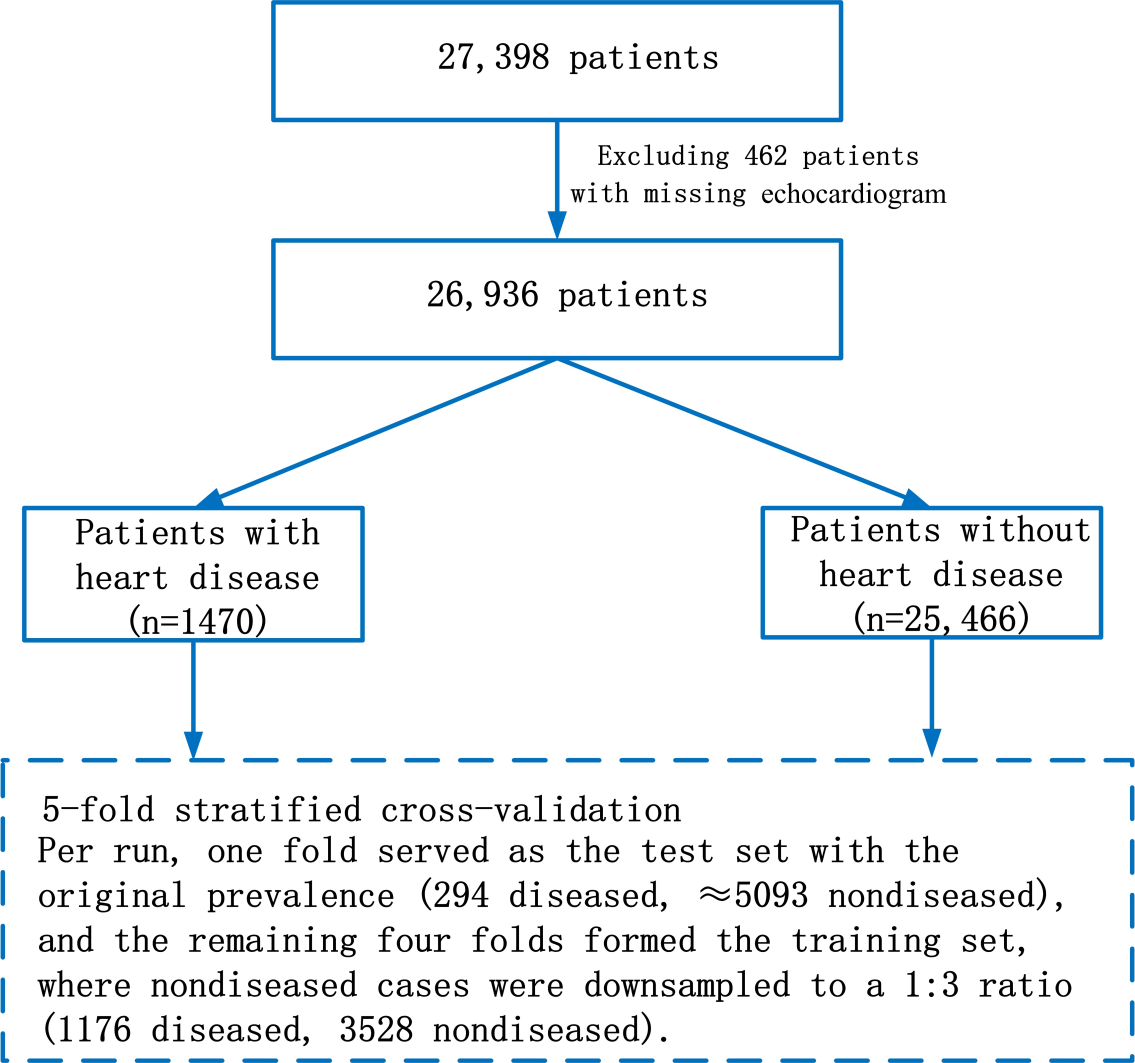
Patient selection flowchart. The flowchart illustrates the selection of 26,936 individuals with paired echocardiogram images and electronic medical records, including 1470 patients with heart disease and 25,466 individuals without HD, from the initial dataset. This entire cohort was used for patient-level stratified 5-fold cross-validation.

To rigorously evaluate performance and avoid data leakage, we adopted a patient-level stratified 5-fold cross-validation scheme on the entire cohort of 26,936 individuals (1470 with HD and 25,466 without HD) as shown in Figure 2. All patients were randomly partitioned into 5 nonoverlapping folds of approximately equal size while preserving the original, imbalanced prevalence of HD; thus, each fold contained 294 patients with HD and approximately 5093 patients without HD. In each cross-validation iteration, one fold was held out as the test set (294 diseased and ≈5093 nondiseased individuals), and the remaining 4 folds were combined as the training set (1176 diseased and ≈20,372 nondiseased individuals). To address class imbalance during model training, we retained all 1176 diseased cases in the training set and randomly downsampled the nondiseased cases to 3528, yielding a 1:3 ratio of diseased to nondiseased individuals in the training subset. The held-out test fold was never resampled—no down-sampling, up-sampling, or other resampling techniques were applied—so that the evaluation always reflected the real-world prevalence. For each patient in the held-out test fold, 5 4-view inputs were generated by repeated random sampling from that patient’s image pool, and the model output one probability for each sampled 4-view set. These 5 probabilities were then averaged to obtain the final patient-level prediction used for evaluation. Model hyperparameters were tuned on the training data using early stopping based on performance on an internal validation subset of the training folds [39], and all performance metrics, including the AUC, were computed on each untouched test fold and then averaged across the 5 folds. In contrast, for the multiclass and subtype-specific experiments, no over-sampling or downsampling was applied across heart disease subtypes. Models for these tasks were trained and evaluated on the natural, imbalanced distribution of subtypes within each fold.

**Figure 3.**
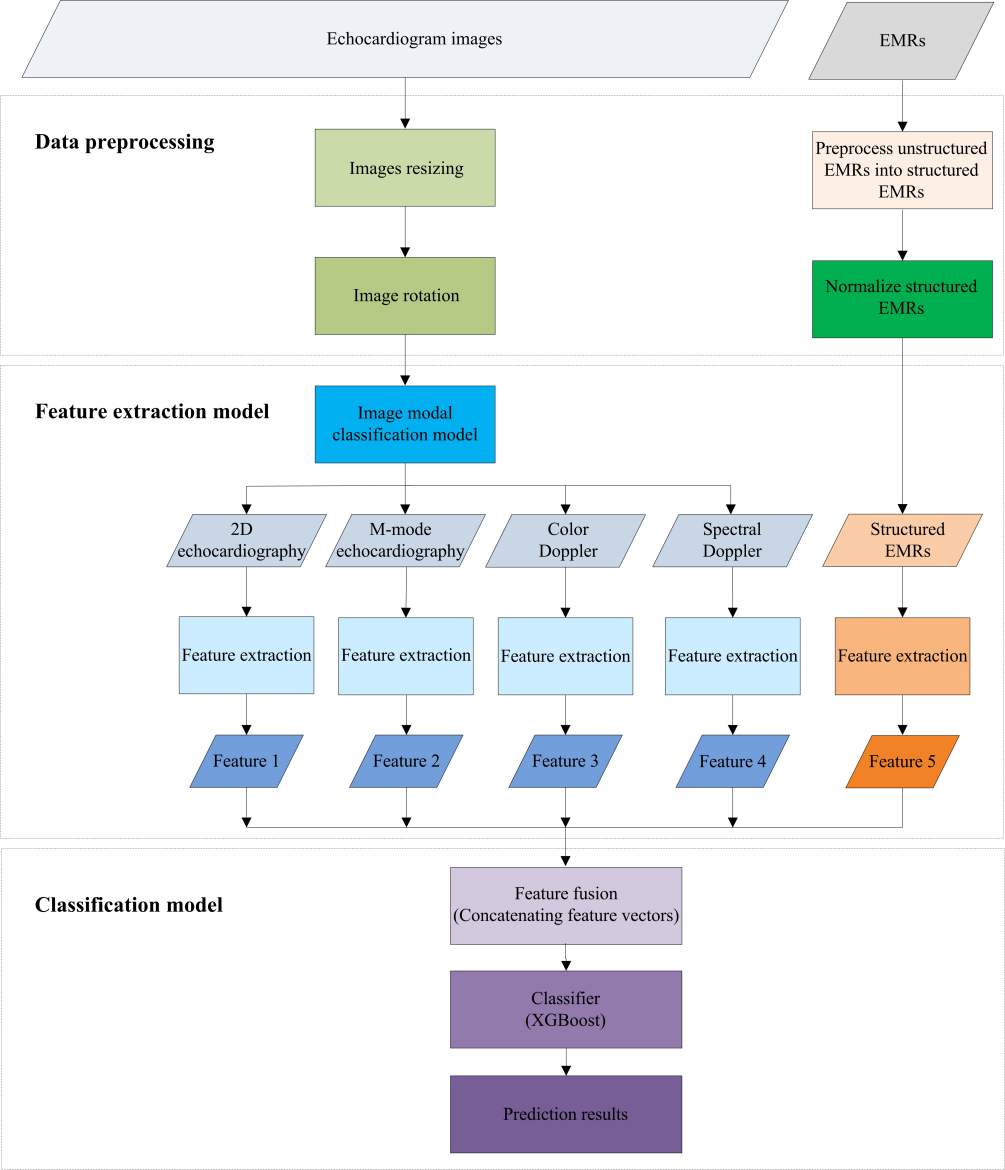
Experimental framework of multimodal classification of heart disease. Four echocardiographic views (2D, M-mode, color Doppler, and spectral Doppler) are processed by simplified Inception-v3 branches to obtain view-specific feature vectors, and electronic medical records are represented by a 12-dimensional clinical feature vector. These five feature vectors are concatenated into a single fused representation, which is then fed into an Extreme Gradient Boosting classifier to produce the final prediction. EMR: electronic medical record; XGBoost: extreme gradient boosting.

Figure 3 provides a detailed schematic of the experimental framework.

The EMRs include 10 structured fields, which are hypertension, DM, hyperlipidemia, hyperuricemia, AR, AF, premature beat, Cpain, Hache, Sex_code, as well as 2 unstructured fields, which are Date_of_birth and Reg_date. For the unstructured field “Date_of_birth” (birthdate), we calculated the difference between it and the date of the last ECHO examination to derive a new field called “age.” For the unstructured field “Reg_date,” which represents the registration date of the first hospital visit, we calculated the difference between this date and the date of the last ECHO examination to obtain a new field called “registration years.” Next, we performed normalization on the final 12 EMR fields, scaling them to a range between 0 and 1.

Each patient’s ECHO image contains 4 views, and these 4 views are mixed together in the original ECHO image data. Initially, we manually classified 2000 ECHO images into 4 view categories. Subsequently, we used this classification result as the training data to develop an AI model based on Inception_V3 for classifying the 4 views. This model is referred to as the ECHO image classifier (UIC). Using the UIC, we classified all ECHO images into their respective 4 views. Due to significant differences between the 4 views, we achieved perfect classification on the annotated test set. This allowed us to effectively separate the ECHO images based on their 4 distinct views. During model training, each input ECHO image is randomly selected from 1 of 4 views. Each person provides up to 16 ECHO images as an image pool, from which 1 image per view is randomly sampled during training to form a 4-view input. During testing and inference, 5 4-view inputs are generated for each patient by repeated random sampling from the same image pool, and the predicted probabilities are averaged to obtain the final patient-level prediction. If a view has fewer than 4 images, subsequent selections from that view will be drawn randomly from previously used images within that view. If a view is completely missing, it is filled with zeros. Notably, all ECHO images fed into the model are resized to 299×299 pixels in advance to fit the model’s input requirements. Additionally, for enhanced model stability, each ECHO image undergoes a random rotation of 1 to 10 degrees before being input into the model.

ECHO images from 4 views and EMRs were used as the 5 inputs to the model. After feature extraction, the 5 corresponding feature vectors were concatenated into a single multimodal representation, which was then fed into an XGBoost classifier to predict the presence or absence of heart disease.

## Results

### Performance of Multimodal Model

We conducted 4 sets of binary classification experiments, including distinguishing between presence or absence of HD, presence or absence of heart valve and blood flow abnormalities, presence or absence of ventricular abnormalities, presence or absence of heart failure. Additionally, we performed 1 set of 3-class classification experiments to differentiate heart valve and blood flow abnormalities, ventricular abnormalities versus heart failure. This 3-class model was trained and evaluated solely on the subset of patients already diagnosed with HD; therefore, no normal or nondisease category is included in this task. From a clinical perspective, this experiment is intended for within-HD coarse phenotyping and triage after disease status has been established, helping prioritize follow-up focus (eg, valvular-focused assessment vs ventricular-function evaluation vs heart-failure–oriented management) and supporting more standardized categorization in clinical workflow. Finally, we conducted 1 set of 4-class classification experiments to distinguish heart valve and blood flow abnormalities, ventricular abnormalities, heart failure versus normal conditions. Detailed view-classifier metrics, per-class multiclass metrics, confusion matrices, precision–recall analysis, and calibration results can be found in Multimedia Appendix 1. These experiments were conducted to comprehensively investigate various aspects of heart health for diagnostic purposes. For all tasks, we evaluated not only our proposed multimodal model but also 2 image-only baseline models, CarpNet and optimized EfficientNetB3 [38]. Both baselines were reimplemented and trained on the same echocardiography dataset as the proposed model and were evaluated under the same patient-level 5-fold stratified cross-validation scheme, using identical train–test folds and echocardiographic inputs to ensure a fair comparison. To assess the effectiveness of the classification models in this study, the main evaluation metric we used is AUC. The receiver operating characteristic curve was generated by plotting the true positive rate against the false positive rate across a range of decision thresholds. In normalized terms, the AUC represents the likelihood that a randomly selected positive instance is assigned a higher score by the classifier than a randomly selected negative instance (under the assumption that positive instances are ranked above negative ones). Consequently, a higher AUC value reflects stronger discriminative ability of the classifier and indicates superior predictive performance of the model. In addition, given the imbalanced nature of the outcome and the intended use as a clinical screening tool, we also report sensitivity, specificity, positive predictive value (PPV), and negative predictive value (NPV) for each model. These metrics are computed at a clinically selected decision threshold (probability cutoff=0.28) on the test dataset. For the 3-class and 4-class tasks, sensitivity, specificity, PPV, and NPV were computed in a one-vs-rest manner for each class, macro-averaged across classes within each held-out test fold, and then summarized as mean± SD across the 5 cross-validation runs. For the binary tasks, all performance metrics were calculated on the held-out test folds and then averaged across the 5 cross-validation runs.

In our study, we conducted 5 sets of binary classification experiments using patient-level stratified 5-fold cross-validation and observed strong overall performance across the binary tasks. Specifically, our model excelled in predicting heart failure with an impressive AUC of 0.9903(SD 0.014), and achieved a substantial AUC of 0.8147 (SD 0.009) in the pivotal task of detecting HD.

Within the 3-class classification experiments, our model demonstrated exceptional performance by effortlessly distinguishing between different subtypes of HDs, achieving an AUC of 0.9914 (SD 0.009). The corresponding macro-averaged threshold-dependent metrics remained high, indicating balanced discrimination across the 3 disease categories.

In the 4-class classification experiments, our model achieved notable AUCs of 0.9298 (SD 0.007) for classifying the 3 subtypes of HD and normal conditions. Because the 4-class task was evaluated by macro-averaging one-vs-rest class-specific metrics under a highly imbalanced class distribution, PPV was lower than sensitivity, whereas specificity remained high across models. The detailed classification results for all binary, 3-class, and 4-class tasks are presented in Table 3.

**Table 3.** Classification results of our proposed multimodal model compared with other models (CarpNet and optimized EfficientNetB3). AUC^a^, sensitivity, specificity, PPV^b^, and NPV^c^ are reported as mean (SD) across 5-fold cross-validation. For the 3-class and 4-class tasks, sensitivity, specificity, PPV, and NPV were computed in a one-vs-rest manner for each class and macro-averaged across classes within each fold. *P* values are obtained from DeLong’s test comparing the AUC of each model with that of the proposed multimodal model.

Classification type	Content	Methods	AUC, mean (SD)	Sensitivity (%), mean (SD)	Specificity (%), mean (SD)	PPV (%), mean (SD)	NPV (%), mean (SD)	*P* value versus ours
2 Classes	Presence - absence of HD^d^	CarpNet	0.7625 (0.014)	76 (2.1)	70.1 (2.4)	12.8 (1.9)	98.1 (0.4)	<.001
		Optimized EfficientNetB3	0.7724 (0.012)	80.7 (1.8)	70.7 (2.2)	13.7 (2)	98.4 (0.3)	<.001
		Ours	0.8147 (0.009)	84.6 (1.6)	72.4 (2)	15 (2.3)	98.8 (0.3)	—^e^
	Presence - absence of heart valve and blood flow abnormalities	CarpNet	0.8497 (0.008)	84.6 (1.7)	78.2 (1.9)	12.8 (1.8)	99.3 (0.2)	.01
		Optimized EfficientNetB3	0.8231 (0.008)	82.6 (1.9)	75.1 (2.1)	11.2 (1.7)	99.1 (0.3)	<.001
		Ours	0.8686 (0.015)	88.4 (1.4)	80.3 (1.7)	14.5 (2)	99.5 (0.2)	—
	Presence - absence of ventricular abnormalities	CarpNet	0.7304 (0.013)	74.2 (2.4)	66.3 (2.3)	3.2 (0.6)	99.4 (0.2)	<.001
		Optimized EfficientNetB3	0.7116 (0.007)	72.2 (2.2)	65.1 (2.1)	3 (0.5)	99.4 (0.2)	<.001
		Ours	0.7573 (0.008)	78.3 (1.9)	68.3 (1.9)	3.5 (0.6)	99.5 (0.2)	—
	Presence - absence of heart failure	CarpNet	0.9734 (0.008)	88.4 (2)	93.9 (1.1)	8.9 (1.6)	99.9 (0.1)	.02
		Optimized EfficientNetB3	0.9746 (0.011)	89.5 (0.8)	94.2 (1)	9.5 (1.7)	99.9 (0.1)	.049
		Ours	0.9903 (0.014)	92.8 (1.4)	94.8 (0.9)	10.8 (1.8)	99.9 (0.1)	—
3 Classes	Heart valve and blood flow abnormalities - ventricular abnormalities - heart failure	CarpNet	0.9726 (0.004)	88.5 (1.4)	94.3 (1)	85.2 (1.6)	92.9 (1.2)	<.001
		Optimized EfficientNetB3	0.9547 (0.015)	86.5 (1.7)	93.3 (1.1)	85.7 (1.5)	93.6 (0.9)	<.001
		Ours	0.9914 (0.009)	94.2 (1)	97.1 (0.7)	92.3 (0.8)	96.0 (0.9)	—
4 Classes	Heart valve and blood flow abnormalities - ventricular abnormalities - heart failure - normal	CarpNet	0.8942 (0.015)	87.6 (1.5)	95.9 (0.8)	46.8 (1.6)	81.9 (1.1)	<.001
		Optimized EfficientNetB3	0.8835 (0.0012)	86.6 (1.6)	95.5 (0.9)	48.6 (1.3)	82.6 (0.8)	<.001
		Ours	0.9298 (0.007)	90.7 (1.2)	96.9 (0.7)	52.3 (1.4)	84.1(0.6)	—

a AUC: area under the receiver operating characteristic curve.

b PPV: positive predictive value.

c NPV: negative predictive value.

d HD: heart disease.

e Not applicable.

### Ablation Study

As shown in Table 4, we observe that our multimodal model can benefit from all modal data, including EMRs and images. The ablation results show that without any data from the 2 data, the performance of the classification results may decline. The last configuration of Table 4 demonstrates that our method achieves the best overall performance with all classification results.

**Table 4.** Ablation study of our proposed multimodal model on HD^a^ using different input modalities. The EMRs^b^ column corresponds to an EMR-only model implemented with XGBoost^c^, the Image column corresponds to an echocardiogram-only CNN^d^ model based on the simplified Inception-V3 architecture, and the Ours column corresponds to the joint EMR-image fusion model. AUC^e^, sensitivity, specificity, PPV^f^, and NPV^g^ are reported as mean (SD) across 5-fold cross-validation. For the 3-class and 4-class tasks, sensitivity, specificity, PPV, and NPV were computed in a one-vs-rest manner for each class and macro-averaged across classes within each fold. *P* values are obtained from DeLong’s test comparing the AUC of each ablation setting with that of the full multimodal model.

Classification type	Content	Methods	AUC, mean (SD)	Sensitivity (%), mean (SD)	Specificity (%), mean (SD)	PPV (%), mean (SD)	NPV (%), mean (SD)	*P* value versus ours
2 Classes	Presence - absence of HD	EMRs	0.7343 (0.009)	77.5 (2.1)	67 (2.3)	11.9 (1.7)	98.1 (0.4)	<.001
		Image	0.7785 (0.014)	82.3 (1.9)	73.5 (2)	15.2 (2.1)	98.6 (0.3)	<.001
		Ours	0.8147 (0.009)	84.6 (1.6)	72.4 (2)	15 (2.3)	98.8 (0.3)	—^h^
	Presence - absence of heart valve and blood flow abnormalities	EMRs	0.7747 (0.008)	80.5 (2)	76.5 (2.1)	11.5 (1.6)	99 (0.3)	<.001
		Image	0.8315 (0.007)	86.6 (1.7)	81.6 (1.8)	15.1 (2)	99.4 (0.2)	<.001
		Ours	0.8686 (0.015)	88.4 (1.4)	80.3 (1.7)	14.5 (2)	99.5 (0.2)	—
	Presence - absence of ventricular abnormalities	EMRs	0.6927 (0.015)	71.2 (2.4)	66.1 (2.3)	3 (0.5)	99.4 (0.2)	<.001
		Image	0.7208 (0.009)	74.7 (2.1)	68.9 (2)	3.4 (0.6)	99.5 (0.2)	<.001
		Ours	0.7573 (0.008)	78.3 (1.9)	68.3 (1.9)	3.5 (0.6)	99.5 (0.2)	—
	Presence - absence of heart failure	EMRs	0.9442 (0.0013)	89 (1.8)	96.2 (0.8)	13.7 (2.1)	99.9 (0.1)	<.001
		Image	0.9762 (0.009)	92.8 (1.5)	97.8 (0.6)	22.2 (2.5)	100 (0)	.06
		Ours	0.9903 (0.014)	92.8 (1.4)	94.8 (0.9)	10.8 (1.8)	99.9 (0.1)	—
3 Classes	Heart valve and blood flow abnormalities - ventricular abnormalities - heart failure	EMRs	0.9539 (0.013)	90.2 (1.5)	95.1 (0.9)	87.8 (1.3)	94.2 (0.8)	<.001
		Image	0.9744 (0.010)	93.1 (1.2)	96.6 (0.8)	90.2 (1.4)	95.7 (0.6)	.005
		Ours	0.9914 (0.009)	94.2 (1)	97.1 (0.7)	92.3 (0.8)	96.0 (0.9)	—
4 Classes	Heart valve and blood flow abnormalities - ventricular abnormalities - heart failure - normal	EMRs	0.8498 (0.010)	86.2 (1.6)	95.4 (0.9)	47.6 (1.3)	82.1 (0.7)	<.001
		Image	0.8835 (0.008)	88.3 (1.4)	96.1 (0.8)	49.4 (1.6)	83.4 (0.9)	<.001
		Ours	0.9298 (0.007)	90.7 (1.2)	96.9 (0.7)	52.3 (1.4)	84.1 (0.6)	—

a HD: heart disease.

b EMR: electronic medical record.

c XGBoost: extreme gradient boosting.

d CNN: convolutional neural network‌.

e AUC: area under the receiver operating characteristic curve.

f PPV: positive predictive value.

g NPV: negative predictive value.

h Not applicable.

### Analysis of Explainability

**Figure 4.**
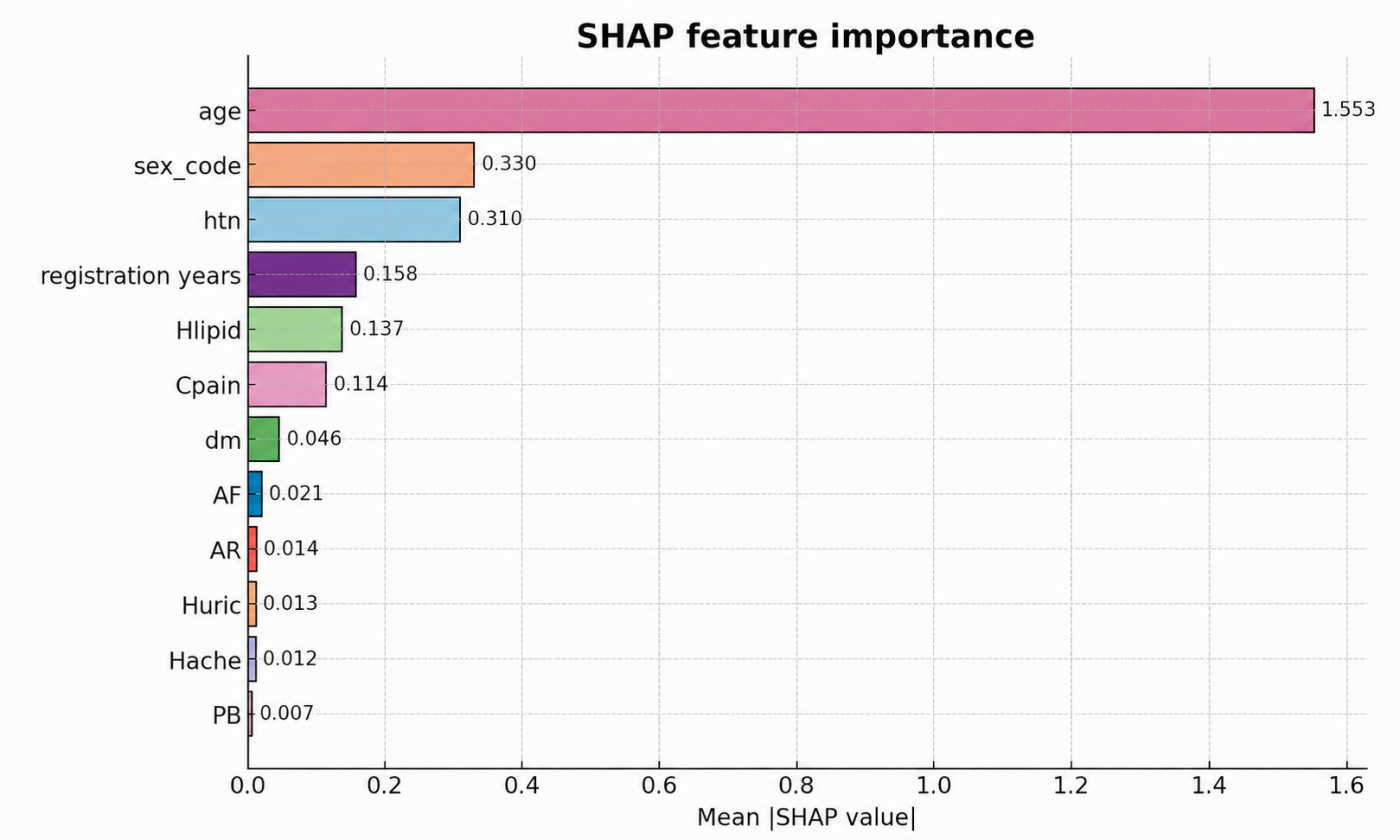
Shapley additive explanations-based global feature importance of electronic medical records variables for the extreme gradient boosting model. Bars represent the mean absolute Shapley additive explanations value of each feature, reflecting its overall contribution to model predictions. Higher values indicate stronger influence. AF: atrial fibrillation; AR: arrhythmias; Cpain: chest pain or tightness; DM: diabetes mellitus; HTN: hypertension; Hlipid: hyperlipidemia; Huric: hyperuricemia; PB: premature beats.

To interpret the contribution of EMR variables to the XGBoost classifier, we adopted SHAP rather than relying on *F* scores, which may be biased toward variables with high cardinality or frequent tree splits. SHAP provides a unified and theoretically grounded method to quantify the marginal contribution of each feature to the predicted risk of heart disease. Figure 4 presents the SHAP-based global feature importance of the EMR variables. Age exhibits the highest contribution, indicating its dominant role in the model’s predictive output. Demographic and cardiovascular risk factors—including sex_code (0.330), hypertension (0.310), and registration years (0.158)—also demonstrate notable influence. Metabolic variables such as hyperlipidemia (0.137) and symptom-related features such as Cpain (0.114) contribute moderately. In contrast, DM, AF, AR, hyperuricemia, Hache, and premature beat show smaller SHAP values, suggesting weaker but still measurable influence in the examined cohort.

**Figure 5.**
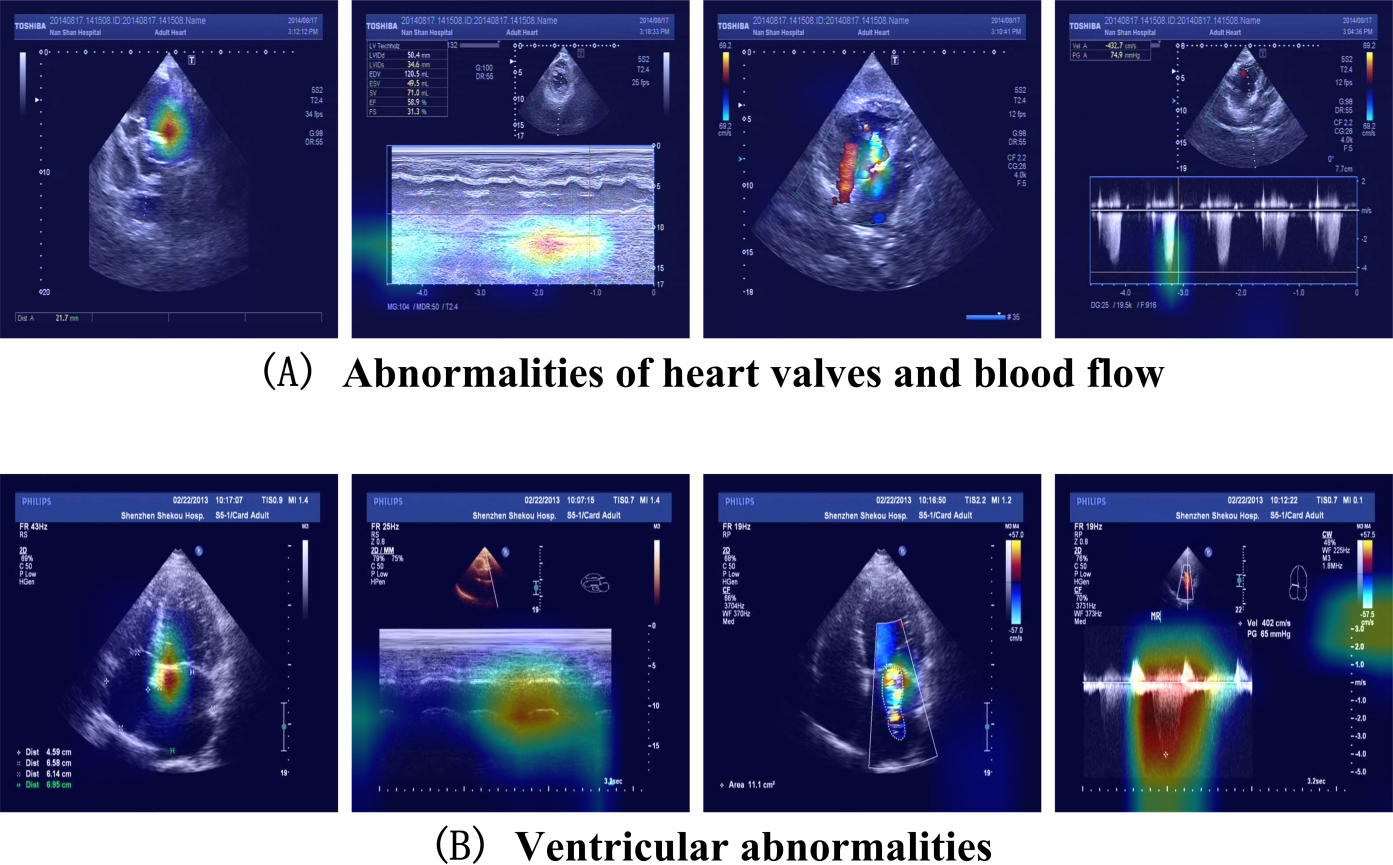
Echocardiogram images’ heatmap. Gradient-weighted class activation mapping (Grad-CAM) was applied to the simplified Inception-v3 branches to highlight image regions that contributed most to the model’s predictions.

The inherent complexity and opaque decision-making processes of machine learning models—particularly neural networks—frequently hinder the interpretability of their predictions. This lack of transparency presents a significant challenge, particularly in health care, where trust in the decision-making process is paramount. To ensure the reliability and usability of these models, it is vital that their inner workings are not only highly accurate but also interpretable to medical professionals. Fortunately, advancements in data visualization techniques have made it easier to understand these complex models. For instance, heatmaps can now be used to highlight areas of focus in a model’s decision-making process, offering clearer insight into how neural networks process data. To interpret the model predictions and visualize which image regions contributed most to the classification, we generated class-discriminative heatmaps using Gradient-weighted Class Activation Mapping (Grad-CAM), as shown in Figure 5. For each echocardiographic view, Grad-CAM was applied to the convolutional feature maps of the simplified Inception-v3 branch corresponding to that view to compute importance weights with respect to the predicted class. The resulting activation maps were then upsampled to the original image resolution and overlaid on the echocardiographic images to highlight regions that were most influential for the model’s decision. These heatmaps were used as a qualitative tool to visualize which regions of the input images contributed most to the model’s predictions, and were inspected by an experienced cardiologist to assess whether the highlighted areas overlapped with clinically relevant cardiac structures and abnormal flow patterns. Figure 5A presents the heatmap of a patient with valvular heart disease, specifically aortic stenosis. In the 2D echocardiography, our model’s attention is focused around the aortic valve, where echo signals are observed. In the color Doppler image, the model highlights a large area of red-blue aliasing. The spectral Doppler image shows an increased blood flow velocity in the left ventricular outflow tract (LVOT). These areas of interest closely align with clinical diagnostic criteria for aortic stenosis. The presence of echoes around the aortic valve suggests possible calcification. The extensive aliasing indicates high-velocity and turbulent flow, which is a typical manifestation of stenosis. Furthermore, the elevated blood flow velocity and the increased peak pressure gradient—characterized by the sharp “dagger-shaped” high-velocity spectral waveform—are classic indicators of aortic stenosis. Figure 5B shows the heatmap of a patient with ventricular abnormality, specifically dilated cardiomyopathy. In the 2D echocardiography, our model focuses on an enlarged left ventricle. The M-mode echocardiography shows that the model attends to a flattened motion pattern of the anterior and posterior walls of the left ventricle, with reduced amplitude. The color Doppler image highlights a mosaic-like turbulent flow region, and the spectral Doppler reveals increased flow velocity. These findings are consistent with clinical features of dilated cardiomyopathy. The left ventricular diameter is measured at 6.95 cm, significantly exceeding the normal upper limit (~5.8 cm, varying slightly by sex), indicating possible dilation. The reduced amplitude of wall motion supports impaired myocardial contractility, suggestive of an underlying myocardial disorder such as dilated cardiomyopathy. The mosaic flow pattern indicates prominent regurgitation, likely due to mitral or tricuspid valve incompetence. This regurgitation may be secondary to annular dilation caused by ventricular enlargement. The peak flow velocity reaches 402 cm/s, with a transvalvular pressure gradient of 65 mmHg, which is characteristic of severe mitral regurgitation commonly observed in dilated cardiomyopathy due to leaflet tethering and annular dilation. The integration of all 4 echocardiographic modalities supports a confident diagnosis of dilated cardiomyopathy. Overall, these Grad-CAM visualizations provide qualitative evidence that the model tends to focus on anatomically and physiologically meaningful regions that clinicians routinely examine when assessing valvular and ventricular abnormalities. However, this analysis remains illustrative and subjective, and we did not perform a formal quantitative comparison with expert-annotated regions or reader studies in this work.

## Discussion

### Principal Findings

This study presents a multimodal deep learning framework that integrates echocardiographic images and EMRs for comprehensive HD screening. The proposed model demonstrated strong discriminative performance across a range of binary and multiclass classification tasks. Notably, the model achieved an AUC of 0.8147 (SD 0.009) in the critical task of detecting HD presence and an AUC of 0.9903 (SD 0.014) in predicting heart failure, supporting its potential clinical relevance in large-scale check-up settings where echocardiography is routinely acquired. Notably, for heart failure, the EMR-only baseline already achieved strong discrimination (AUC 0.9442, SD 0.011), suggesting that routine clinical variables are highly predictive in this cohort. The multimodal model further improved performance (AUC 0.9903, SD 0.014), but the incremental benefit of adding imaging should be interpreted in light of the additional computational cost and workflow integration required for processing echocardiographic data. The ablation study suggests that the benefit of fusion is task-dependent: combining ECHO and EMR features generally improved performance across endpoints, yet for certain outcomes (eg, heart failure), the image-only model was not statistically distinguishable from the multimodal model (*P*=.06). This indicates that fusion may offer limited incremental discrimination when 1 modality already captures most predictive signal or when performance approaches a ceiling. Furthermore, visualization techniques such as heatmaps and feature importance rankings enhanced the model’s interpretability, highlighting clinically relevant inputs such as age, registration time, and hypertension history. These findings suggest that the proposed multimodal approach can augment HD risk stratification and facilitate early intervention, particularly in settings where echocardiography is already routinely acquired (eg, large-scale check-up programs), so that the primary additional burden is computational inference rather than new data collection.receiver operating characteristic curve

### Limitations

Several limitations should be acknowledged.

#### Class Imbalance

For the primary binary screening task (presence vs absence of HD), we used patient-level stratified 5-fold cross-validation and randomly down-sampled non-HD cases within the training folds to a 1:3 HD: non-HD ratio, while preserving the original prevalence in all held-out test folds. This choice improved training tractability and reduced majority-class dominance, but it inevitably discards a portion of non-HD cases and may underuse negative-class diversity. Data-efficient alternatives (eg, class-weighted loss or focal loss) that retain all non-HD cases may further improve robustness and should be examined in future work. For multiclass and subtype-specific tasks, no oversampling or down-sampling was applied; thus, performance on rare subtypes may be less stable.

#### Single-Center Retrospective Design

All data were obtained from 1 medical institution in China. Imaging protocols, patient demographics, and clinical workflows may differ across centers; external multicenter validation is required before deployment.

#### Explainability Validation

Although Grad-CAM and SHAP were used to improve interpretability, the current explainability assessment remains largely qualitative and may be subjective. In particular, the alignment between model-highlighted regions and physiological pathology has not been statistically quantified. We did not collect expert-annotated regions of interest (ROIs) or conduct reader studies; therefore, localization agreement metrics (eg, Intersection-over-Union [IoU] or Dice overlap with radiologist-annotated ROIs) were not measured.

#### Modeling, Index Date Definition, and EMR Feature Coding Assumptions

EMR information was summarized into cross-sectional features (eg, age and registration years) rather than modeling full longitudinal trajectories. In addition, the “current date” used for feature engineering was defined as the date of the last echocardiographic examination; thus, for patients with multiple visits, the analysis is anchored to the final time point. This may bias the cohort toward later or more established disease and may limit generalizability to early-stage screening settings where only earlier visits are available. In addition, binary clinical history and symptom variables were coded as 1 if documented and 0 otherwise; in retrospective EMRs, documentation may be incomplete (ie, underreporting), so this assumption may introduce systematic bias.

#### View Classification Generalization

Although the view classifier achieved perfect classification on the annotated test set, real-world echocardiography acquisitions may include off-axis or incomplete views, variable probe positioning, atypical protocols, motion artifacts, and reduced image quality. These factors can introduce view ambiguity (especially between neighboring views) and may lead to misclassification when deployed in routine clinical workflows.

#### Prospective Use

The model has not yet been evaluated in real-time clinical workflows; prospective studies are needed to assess clinical use and workflow integration.

### Future Directions

Future work will focus on external multicenter validation and prospective evaluation in real-world workflows. We will also investigate data-efficient imbalance handling (eg, class-weighted loss and/or focal loss trained on all available non-HD cases) and assess their impact on discrimination, calibration, and robustness. To strengthen explainability, we plan to collect expert-annotated regions of interest (ROIs) for representative pathological patterns and to quantitatively evaluate attention–pathology alignment using localization agreement metrics such as Intersection-over-Union (IoU) and/or Dice overlap, potentially complemented by reader studies. In addition, we will explore more fine-grained subtype modeling, incorporate longitudinal EMR trajectories, and further improve probability reliability through post-hoc calibration and threshold optimization to balance sensitivity and screening burden in practical deployment.

### Conclusions

In this study, we developed a multimodal framework that integrates echocardiography and EMR features for HD screening and category-level classification in large-scale health check-up settings.

Across the primary HD detection task (HD vs non-HD) and subsequent category-level multiclass tasks, the multimodal model achieved favorable discriminative performance and outperformed image-only baselines in our experiments, indicating that combining echocardiography with structured clinical variables can improve screening performance compared with using a single modality.

To support model transparency, we applied Grad-CAM to visualize image regions contributing to predictions and used SHAP to quantify the relative importance of key EMR features, providing complementary qualitative interpretability signals for clinical review.

## Supplementary material

10.2196/78949 Multimedia Appendix 1 Supplementary evaluation results for the proposed model, including additional classification analyses and reliability assessments: view separation classifier protocol and performance (Table S1); imbalance-sensitive multiclass per-class metrics and confusion matrices for the 3-class and 4-class tasks (Tables S2–S5); precision–recall curve for HD vs non-HD risk prediction (Figure S1); and probability calibration (reliability) curve with calibration metrics and related sensitivity analyses (Figure S2; Tables S6-S7).

## Abbreviations

AF: atrial fibrillation
AI: artificial intelligence
AUC: area under the receiver operating characteristic curve
Cpain: chest tightness or pain
DM: diabetes mellitus
ECHO: echocardiogram
EMR: electronic medical record
HD: heart disease
NPV: negative predictive value
PPV: positive predictive value
SHAP: Shapley additive explanations
XGBoost: extreme gradient boosting
