# Supplementary material for: Multimodal Fusion of Echocardiogram Images and Electronic Medical Records for Heart Disease Screening: Retrospective Algorithm Development and Validation Study
Source: JMIR Med Inform. 2026 May 14;14:e78949. doi: 10.2196/78949 (PMC13175451; doi:10.2196/78949)
Supplement: Multimedia Appendix 1 [file medinform-v14-e78949-s001.doc]

For automatic view separation, we manually labeled approximately 2,000 echocardiographic frames into four categories (2D, M-mode, Color Doppler, and Spectral Doppler). These categories exhibit large and distinctive visual differences, including grayscale vs. black–white line-tracing patterns, presence of a multicolor velocity scale bar, and the appearance of spectral waveforms in Spectral Doppler images. To avoid information leakage, we performed the train–test split at the patient ID level, ensuring that frames/images extracted from the same patient (and the same echocardiography acquisition/video) were assigned exclusively to either the training set or the held-out test set. The dataset was split into 80% for training (≈1,600 images) and 20% for testing (402 images).

The classifier achieved perfect classification on the annotated test set. The confusion matrix was perfectly diagonal, with class-wise precision, recall, and F1-scores all equal to 1.000. Detailed metrics are provided in Table S1.

**Table S1. Performance of the view classifier on the held-out test set.**

| Categories | Precision | Recall | F1-score | Support |
| --- | --- | --- | --- | --- |
| 2D echocardiography | 1.0000 | 1.0000 | 1.0000 | 171 |
| M-mode echocardiography | 1.0000 | 1.0000 | 1.0000 | 35 |
| color Doppler | 1.0000 | 1.0000 | 1.0000 | 87 |
| spectral Doppler | 1.0000 | 1.0000 | 1.0000 | 109 |

To provide an imbalance-sensitive evaluation for the multiclass setting, we report per-class precision, recall (sensitivity), and F1-score for each category, together with the corresponding confusion matrices (counts). Specifically, Appendix Table S2 summarizes per-class metrics for the 3-class task (heart valve and blood flow abnormalities vs ventricular abnormalities vs heart failure), and Appendix Table S3 reports the associated 3×3 confusion matrix. Appendix Table S4 summarizes per-class metrics for the 4-class task (heart valve and blood flow abnormalities vs ventricular abnormalities vs heart failure vs normal), and Appendix Table S5 reports the associated 4×4 confusion matrix. These results complement the AUC-based analysis by explicitly showing class-wise performance and misclassification patterns. The multiclass sensitivity, specificity, PPV, and NPV reported in the main manuscript tables were derived from one-vs-rest class-specific calculations and macro-averaged across classes within each cross-validation fold.

**Table S2.** Per-class precision, recall, and F1-score for the 3-class task (heart valve and blood flow abnormalities vs ventricular abnormalities vs heart failure).

| Subtype | Precision | Recall | F1-score |
| --- | --- | --- | --- |
| Heart valve and blood flow abnormalities | 0.9731 | 0.9425 | 0.9576 |
| Ventricular abnormalities | 0.8868 | 0.9183 | 0.9023 |
| Heart failure | 0.9086 | 0.9890 | 0.9471 |
| Macro (average) | - | - | 0.9356 |

**Table S3.** Confusion matrix (counts) for the 3-class task (heart valve and blood flow abnormalities vs ventricular abnormalities vs heart failure).

| True \ Pred | Heart valve and blood flow abnormalities | Ventricular abnormalities | Heart failure |
| --- | --- | --- | --- |
| Heart valve and blood flow abnormalities | 869 | 42 | 11 |
| Ventricular abnormalities | 23 | 337 | 7 |
| Heart failure | 1 | 1 | 179 |

**Table S4.** Per-class precision, recall, and F1-score for the 4-class task (heart valve and blood flow abnormalities vs ventricular abnormalities vs heart failure vs normal).

| Subtype | Precision | Recall | F1-score |
| --- | --- | --- | --- |
| Heart valve and blood flow abnormalities | 0.343 | 0.921 | 0.500 |
| Ventricular abnormalities | 0.385 | 0.883 | 0.536 |
| Heart failure | 0.367 | 0.901 | 0.522 |
| Normal | 0.996 | 0.907 | 0.950 |
| Macro (average) | - | - | 0.627 |

**Table S5.** Confusion matrix (counts) for the 4-class task (heart valve and blood flow abnormalities vs ventricular abnormalities vs heart failure vs normal).

| True \ Pred | Heart valve and blood flow abnormalities | Ventricular abnormalities | Heart failure | Normal |
| --- | --- | --- | --- | --- |
| Heart valve and blood flow abnormalities | 849 | 7 | 8 | 58 |
| Ventricular abnormalities | 8 | 324 | 21 | 14 |
| Heart failure | 4 | 2 | 163 | 12 |
| Normal | 1611 | 508 | 252 | 23095 |

To address the practical utility of risk estimates for the primary HD detection task, we additionally report the precision–recall (PR) curve and probability calibration results for the HD vs non-HD classification. Figure S1 shows the PR curve and highlights the operating point used in the main results (Recall = 0.846, Precision/PPV = 0.150 and cutoff = 0.28). This curve makes explicit the screening trade-off: operating at a high-recall point increases the number of detected HD cases but inevitably reduces precision (PPV), leading to more false-positive referrals, whereas moving the threshold to the left along the curve improves precision at the cost of lower sensitivity. Therefore, Figure S1 provides an operational view for threshold selection depending on whether the clinical workflow prioritizes minimizing missed cases (high recall) or reducing unnecessary follow-up (higher precision).

**Figure S1.** Precision–recall curve for HD vs non-HD risk prediction.


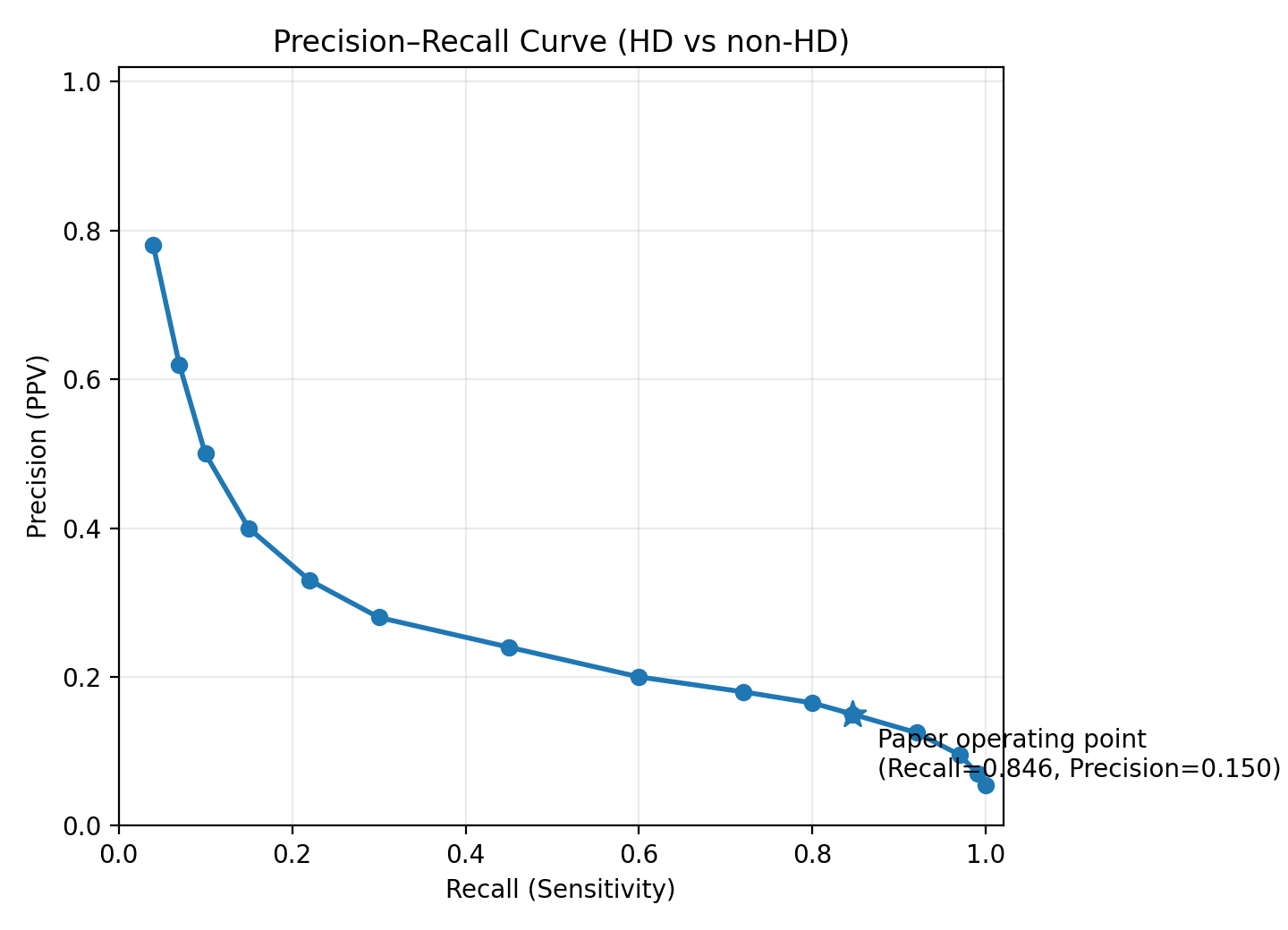


Figure S2 presents the calibration (reliability) curve, comparing mean predicted probabilities with observed event rates across probability bins. Points close to the diagonal indicate that predicted probabilities are well aligned with empirical risk, supporting the interpretation of model outputs as risk estimates for risk stratification. Deviations from the diagonal reflect miscalibration, implying that predicted probabilities may be over- or under-confident in certain ranges. In our results, calibration is generally more informative in the mid-to-high probability region, suggesting that the model’s risk scores can separate higher-risk individuals, while some deviation remains at lower probability bins, indicating potential room for improvement via post-hoc calibration if strict probability accuracy is required for deployment.

**Figure S2.** Calibration curve (reliability diagram) for HD vs non-HD risk prediction.


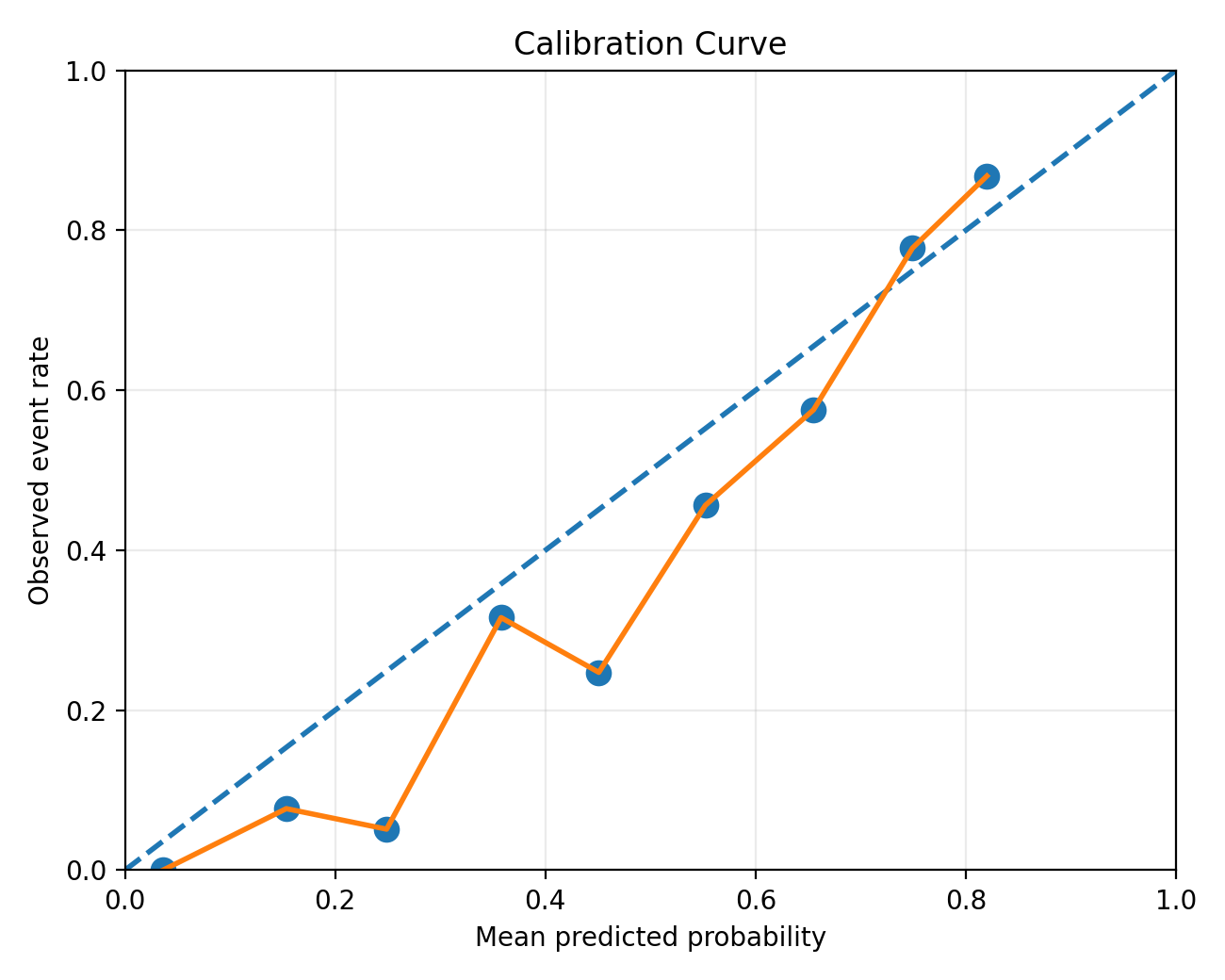


Table S6 quantifies the reliability of the predicted probabilities. The ECE (0.0769) indicates a moderate calibration error (≈7–8% average absolute gap between predicted and observed risks across bins), and the Brier score (0.1856) further suggests non-negligible probabilistic error. The negative intercept (-0.4736) together with a slope >1 (1.4581) implies systematic miscalibration: the model tends to overestimate risk overall, and the probability scale would benefit from post-hoc recalibration. Therefore, the current scores are suitable for risk ranking/triage, while strict probability-based decision making would require calibration on a deployment-matched validation set.

**Table S6.** Calibration metrics for HD^a^ vs non-HD risk prediction.

| Brier score | ECE^a^ (10 bins) | Calibration intercept | Calibration slope |
| --- | --- | --- | --- |
| 0.1856 | 0.0769 | -0.4736 | 1.4581 |

^a^HD: Heart Disease

^b^ECE: Expected Calibration Error‌

To assess whether the feature registration years introduces bias against new patients in a primary screening context, we performed a sensitivity analysis on patients with short registration histories (registration years ≤ 1). We re-evaluated the EMR-only models on this subset using the same evaluation protocol. As shown in Table S7, model performance decreased only modestly compared with the overall test set, suggesting that the predictive performance is not driven primarily by long-term healthcare-system tenure. This analysis provides additional evidence that the proposed approach remains applicable to patients with limited prior EMR history, while also highlighting that probability-based screening decisions may still benefit from calibration and threshold selection tailored to deployment settings.

**Table S7.** Sensitivity analysis on patients with short registration history (registration years ≤ 1). AUC^a^ , sensitivity, specificity, PPV^b^, and NPV^c^ are reported as mean±SD^d^.

| Methods | Dataset | AUC | Sensitivity  （%） | Specificity  （%） | PPV  （%） | NPV  （%） | p-value vs  Overall test set |
| --- | --- | --- | --- | --- | --- | --- | --- |
| EMRs^e^ | registration years≤1 | 0.7240  ± 0.010 | 76.7  ± 2.3 | 66.0  ± 2.2 | 11.5  ± 1.5 | 98.0  ± 0.3 | 0.444 |
| EMRs | Overall test set | 0.7343  ± 0.009 | 77.5  ± 2.1 | 67.0  ± 2.3 | 11.9  ± 1.7 | 98.1  ± 0.4 |  |

^a^AUC: Area Under Curve

^b^PPV: Positive Predictive Value

^c^NPV: Negative Predictive Value

^d^SD: Standard Deviation

^e^EMRs: Electronic Medical Records
